# Recent Trends in Advanced Glycation End Products in Foods: Formation, Toxicity, and Innovative Strategies for Extraction, Detection, and Inhibition

**DOI:** 10.3390/foods13244045

**Published:** 2024-12-14

**Authors:** Shubham Singh Patel, Aarti Bains, Minaxi Sharma, Ankur Kumar, Baskaran Stephen Inbaraj, Prince Chawla, Kandi Sridhar

**Affiliations:** 1 Department of Food Technology and Nutrition, School of Agriculture, Lovely Professional University, Phagwara 144411, Punjab, India; 2 Department of Microbiology, Lovely Professional University, Phagwara 144411, Punjab, India; 3 Research Centre for Life Science and Healthcare, Nottingham Ningbo China Beacons of Excellence Research and Innovation Institute (CBI), University of Nottingham Ningbo China, Ningbo 315100, China; 4 Department of Interdisciplinary Sciences, National Institute of Food Technology Entrepreneurship and Management, Sonipat 131028, Haryana, India; 5 Department of Food Science, Fu Jen Catholic University, New Taipei City 24205, Taiwan; 6 Department of Food Technology, Karpagam Academy of Higher Education (Deemed to be University), Coimbatore 641021, Tamil Nadu, India

**Keywords:** advanced glycation end products, food safety, food toxicity, detection, inhibition, health effects

## Abstract

Advanced glycation end products (AGEs) are produced in foods during their thermal treatment through routes like the Maillard reaction. They have been linked to various health issues such as diabetes, neurodegenerative disorders, and cardiovascular diseases. There are multiple pathways through which AGEs can form in foods and the body. Therefore, this review work aims to explore multiple formation pathways of AGEs to gain insights into their generation mechanisms. Furthermore, this review work has analyzed the recent trends in the detection and inhibition of AGEs in food matrices. It can be highlighted, based on the surveyed literature, that UHPLC-Orbitrap-Q-Exactive-MS and UPLC-ESI-MS/MS can produce highly sensitive results with a low limit of detection levels for AGEs in food matrices. Moreover, various works on inhibitory agents like spices, herbs, fruits, vegetables, hydrocolloids, plasma-activated water, and probiotic bacteria were assessed for their capacity to suppress the formation of AGEs in food products and simulation models. Overall, it is essential to decrease the occurrence of AGEs in food products, and future scope might include studying the interaction of macromolecular components in food products to minimize the production of AGEs without sacrificing the organoleptic qualities of processed foods.

## 1. Introduction

Food processing is an important component of converting raw agricultural produce into various food products, which offers consumers an array of diversified products [1]. Processing raw food materials not only increases palatability but also increases the bioavailability of some macro-nutrients such as proteins and carbohydrates. Furthermore, processing also aids in shelf-life extension, the production of characteristic flavor and texture, and improving the overall nutritional profile of food products [2]. However, under particular processing conditions (like thermal processing), some compounds might be produced in foods that have been studied for imparting deleterious health effects. For instance, protein-rich and carbohydrate-containing foods may undergo chemical reactions like the Maillard reaction (MAR) during thermal treatments and even during storage. It is a non-enzymatic browning reaction between reducing sugars and various amino sources. MAR involves three steps, i.e., the initial, intermediate, and final. During the initial stage, the formation of Schiff base occurs through the reaction between a free amino group (such as from an amino acid) and a carbonyl moiety (like from a reducing sugar) with the release of water molecules [3]. The second intermediate stage includes sugar dehydration, sugar fragmentation, and Strecker degradation, where pale yellow or colorless products are products. The final stage of MAR comprises aldol condensation, aldehyde–amine polymerization, and the formation of heterocyclic nitrogenous compounds with the formation of brown-colored products called melanoidins. However, the production, involvement, and interaction of other compounds like dicarbonyls can lead to the formation of well-studied compounds like advanced glycation end products, which have been discussed thoroughly in this review work. 

Advanced glycation end products or AGEs can be grouped into two classes, i.e., exogenous and endogenous. The formation of endogenous AGEs is directly related to normal metabolic processes, where the interactions between reducing sugars and proteins or amino groups can lead to their accumulation in the tissues and body fluids [4]. If this accumulation exceeds safe levels, the high concentration can turn virulent for the host body. These compounds have been studied for their ability to induce reactive oxygen species (ROS) and reactive nitrogen species (RNS) production, which are linked with the formation of protein aggregates, cellular abnormalities, apoptosis, and organ dysfunction. Dietary AGEs (type of exogenous AGEs), on the other hand, can form in processed food products like bakery products, processed meats, ice cream, and soft drinks that consist of sugars like corn syrup (containing high quantities of fructose) and are produced in higher amounts in comparison to their endogenous counterparts [5].

Studies have also shown AGEs’ capacity to cause neuro-degradative conditions like Alzheimer’s diseases (AD). However, they are more well known for their role in aggrandizing health-related complications including cardiovascular issues, acting as mediators in increasing obesity risk, and driving insulin resistance leading to type 2 diabetes mellitus [6]. Thus, the detection of these harmful substances is important as thermally processed food products occupy a huge piece of the food industry, but there are no concrete studies on what the minimum safe threshold for their consumption should be. 

Their detection has been made possible using techniques like liquid chromatography, which has been successfully used for measuring AGEs in food samples. The UHPLC variant of the liquid chromatography technique combined with an Orbitrap mass spectrometer was used to detect 15 AGEs [7]. Various inhibitory agents like spices, fruits, vegetables, herbs, and probiotic bacteria have been discussed in this work to expand on their impact on AGE formation during heat treatment or cooking processes like roasting and steaming. To the best of our knowledge, no other review work aimed at AGEs has discussed the role of hydrocolloids and other advanced approaches like plasma-activated water in inhibiting AGEs formation in food products. 

Therefore, the purpose of this review was to highlight and describe the toxic nature of AGEs by surveying and reviewing multiple studies and providing an overview of their formation through various pathways. Furthermore, the recent trends in their extraction and detection with a discussion on various chromatographic techniques employed in their enumeration in food matrices were reviewed. Moreover, we explored several possible routes for their inhibition, such as spices, herbs, fruits, vegetables, and hydrocolloids for their alleviation in heat-processed foods. This review will provide an overview of the recent trends observed in the extraction, detection, and inhibition of AGEs in heat-treated food products.

## 2. Formation of AGEs

The formation of important flavoring compounds like Strecker aldehydes, furans, pyrazines, and thiopenes occurs from the reactive dicarbonyls (such as methylglyoxal and 3-deoxyglucosone) that develop from the interaction of the Amadori rearrangement products (ARPs), Schiff base, and sugars. These dicarbonyls also take part in the formation of stable AGEs. Therefore, there are multiple constituents involved in the formation of AGEs and their specific pathways leading to their generation. Thus, understanding these pathways is imperative to gain insights into the formation of AGEs (endogenous and exogenous).

**Figure 1:**
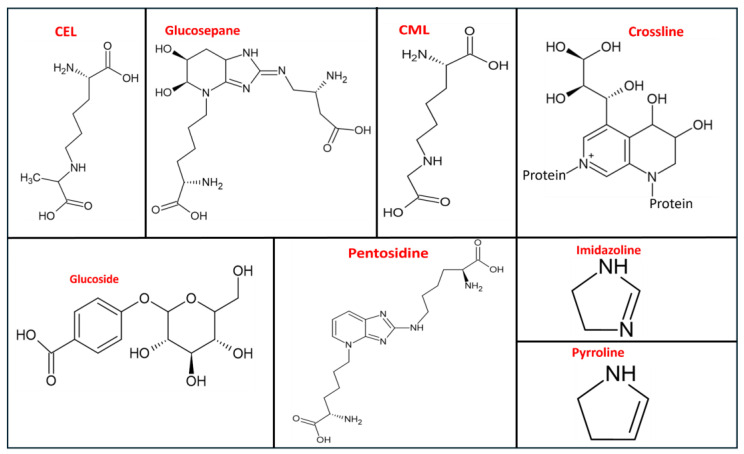
Chemical structures of some advanced glycation end products.

**Figure 2:**
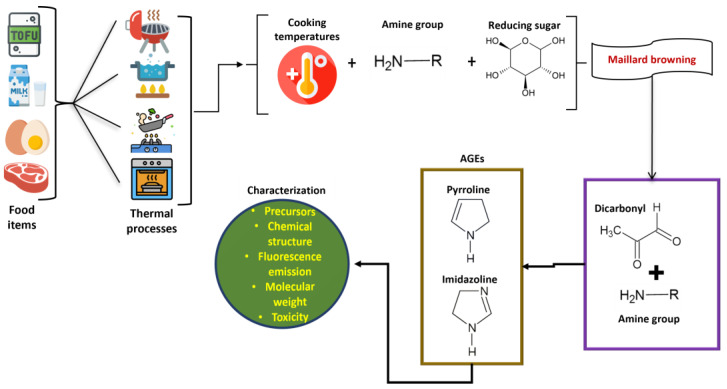
An outline of the formation of advanced glycation end products (AGEs) during thermal cooking processes.

More than 20 AGEs have been discovered from the analysis of proteins present in tissues, which encompass pyrroline, imidazolines, glucosepane, pentosidine, carboxymethyl lysine (CML), carboxyethyl lysine (CEL), crossline, and glucoside (Figure 1) [8]. The occurrence of endogenous AGEs initiates with the non-enzymatic reaction of glycation, which proceeds from a reducing sugar or reactive oxoaldehyde reacting with lipids or amine-rich sources like proteins or nucleic acids (Figure 2). AGEs are the products of the Maillard reaction, requiring reactions between reducing sugars and a free amine by forming a permanent covalent bond. However, lipid oxidation products can also participate in the reaction with amines like free amino groups present in proteins, their phospholipid derivatives, or nucleic acids. The endogenous AGEs follow various pathways (excluding the non-enzymatic Maillard browning reaction), which include the Hodge pathway and the Namiki pathway (in addition to lipid peroxidation), the Wolff pathway of monosaccharide autoxidation, and the polyol pathway [9]. Reactive dicarbonyls, which are the necessary precursors for AGEs, are constantly formed through normal metabolism in the body, and their continuous formation might lead to an excess accumulation of AGEs in the blood [10]. The reaction mechanism involves the Amadori products’ degradation during chemical stages like dehydration and oxidation, which is essential for the production of reactive carbonyl and dicarbonyl molecules such as glyoxal (GO), methylglyoxal (MG), and deoxyglucosone (DG). These dicarbonyl species may also be generated via the Namiki pathway through the decomposition of the covalently connected ARP precursors or Schiff base. These dicarbonyls, in turn, trigger the initiation of advanced glycation with the subsequent formation of some well-known AGEs like N-ε-(carboxymethyl) lysine (CML) and N-ε-(carboxyethyl) lysine (CEL). The formation of CML can also be expressed with certain proposed pathways, such as through the Namiki pathway (the condensation reaction between nucleophilic bonding sites situated on proteins and peptides with free glyoxal) and the Hodge pathway (the cyclization, dehydration, oxidation, and fragmentation of ARPs). 

**Figure 3:**
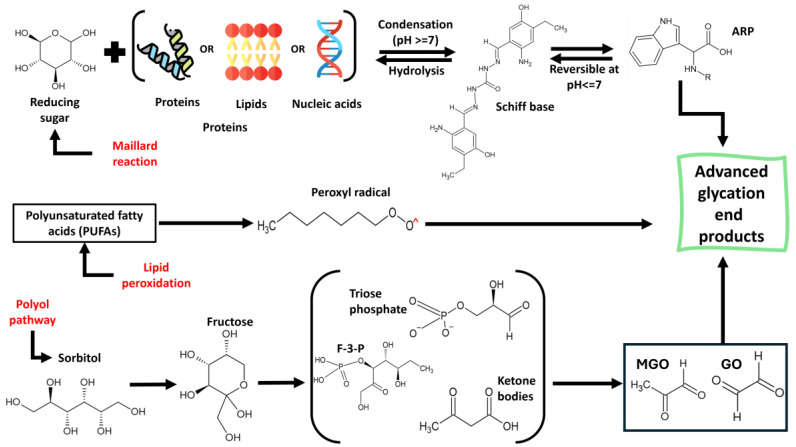
Various pathways for the formation of AGEs. MGO, methylglyoxal; GO, glyoxal; MTCA, (1R,3S)-1-methyl-1,2,3,4-tetrahydro-ß-carboline-3-carboxylic acid; THCA, tetrahydrocannabinolic acid; F-3-P, fructose-3-phosphate.

In contrast to the non-enzymatic reactions, the polyol pathway involves the catalyses of glucose into sorbitol with NADPH acting as the electron donor, which is then oxidized into fructose as the byproduct. It is further phosphorylated into fructose-3-phosphate and finally degraded into a potent AGEs’ precursor 3-deoxyglucosone (3-DG) (Figure 3). Hyperglycemic conditions can accelerate the polyol process of endogenous AGEs’ formation, where sorbitol may get converted to fructose via oxidation by the action of sorbitol dehydrogenase. 

Human physiology is also susceptible to exogenous AGEs through means like modern Western dietary habits and pollutants present in the environment [10]. The dietary AGEs (dAGEs), an extension of exogenous AGEs, are food-derived and developed in foods and food products via the Maillard reaction (as a part of high-heat cooking processes) and have been recorded for their ability to alter gut microflora, affecting the gastrointestinal integrity. 

The process of the dAGEs’ generation happens through the Maillard reaction, which takes place in three stages. The initiation stage includes the generation of a covalent linkage between the amino groups and carbonyl groups, resulting in the formation of an unstable and revertible Schiff base (SB). The SB produces ARPs while undergoing the Amadori rearrangement. Depending on the pH, 1,2-enolization can occur (pH ≤ 7), where the produced ARPs get converted to HMF and furfural. Otherwise, at pH ≥ 7, the ARPs go through 2,3 enolization, where they are converted into reduced ketones (RKs) via deamination, then the RKs undergo a reversible reaction to generate dihydroxyl reduced ketones (DRKs) and further generate nitrogen-free polymers and aldehydes or undergo pyrolysis to directly produce aldehydes. At elevated temperatures and pH ≥ 7, some intermediates such as diacetyl, methylglyoxal (MG), and glyoxal (GO) are formed (Figure 3). The final stage envelopes the reaction of the intermediate reactive dicarbonyl compounds formed during the second stage with free amine groups, or even lipids, with additional cyclization to yield stable AGEs. Considering the diverse pathways through which AGEs can form exogenously and endogenously, it is essential to study the mechanics of their generation as exploring the possible suppression of their formation is quite complex due to the participation of various precursors of varying chemical nature. 

## 3. Toxicological Aspects of AGEs

**Figure 4:**
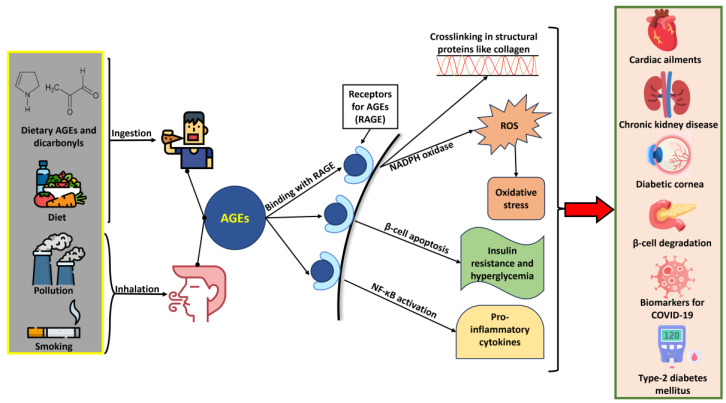
Multiple health effects and conditions induced on exposure to AGEs.

The elevated presence of AGEs in the human body might be taken as an indication of the onset of diseases like Alzheimer’s, atherosclerosis, and diabetes (Figure 4 and Table 1) [11]. The endogenous AGEs are the results of the metabolic processes that include oxidative and non-oxidative pathways, which involve reactions between sugars and heterogenous intermediates like GO and MG. The presence of dAGEs, in contrast, is a direct indication of an individual’s dietary habits and might get assimilated into the body via intestinal absorption. There are two proposed ways through which AGEs may impart their deleterious effects on the body, i.e., protein disruption by intermolecular and intramolecular crosslinking, and interactions with cell receptors that may trigger pathogenic signaling for conditions like diabetes mellitus to surface. 

AGEs promote crosslinking in structural proteins like collagen through the close fibril residues, which form divalent crosslinks and weaken the mechanical properties of tissues by altering their enzymatic crosslinks. The crosslinks result in protein aggregation, which further leads to cellular damage and dysfunctions. AGE-mediated crosslinks in collagen are highly stable and unaffected by the enzymatic correction systems during hyperglycemia and type-2 diabetes, which can lead to diabetic cornea [17]. AGE-induced crosslinks have been related to cardiac issues such as diabetic myocardium (DM). Such conditions occur from the hardening of the myocardial extracellular matrix due to crosslinks formed between matrix proteins catalyzed by AGEs. Some other moderators of diabetes-related cardiac problems have been discovered, such as trimethylamine N-oxide, which can act as a catalyst for the AGE-induced crosslinking of collagen fibers. Various studies have focused on finding biomarkers to trace the onset of cardiovascular risks during diabetes mellitus, which led to the utilization of techniques like skin autofluorescence (SAF) [18]. SAF is a rapid and non-invasive method for determining the target AGE accumulation in tissues for predicting illness development (like cardiovascular issues) before they reach a chronic stage. 

AGEs can also cause a host of health problems by binding with receptors, which can create a disbalance in metabolic processes. Receptors with such affinities are termed RAGEs (receptors for AGEs), which are known for their role as intermediates in bringing complications arising from diabetes [19]. An aggravation of the symptoms during diabetes mellitus may take place by AGEs when they attach to the endothelial cell superficial receptors (RAGEs), leading to an excess formation of ROS by the activation of NADPH oxidase. The formed AGE–RAGE axis has been recognized as a trigger for inflammatory factors like NF-κB (nuclear factor kappa-light-chain-enhancer of activated B cells) and cytokines during diabetic conditions [20]. Diabetic complications arising from the accumulation of AGEs can be attributed to the expression of RAGE in the pancreas, as its activation can result in the apoptosis of insulin-secreting β-cells [21]. The AGE–RAGE axis may also increase the exposure to oxidative/inflammatory tissue damage among overweight/obese children by enhancing oxidative homeostasis dysregulation [22]. THE AGE–RAGE interaction has also been credited for the development of chronic kidney disease (CKD) due to the high production and accumulation of AGEs and depleting kidney functioning [23]. 

Another form of receptor has also been deemed to combine with AGEs, i.e., sRAGE (soluble receptors for AGEs). It is an isoform and a product of proteolytic cleaving of RAGE, which is found in human serum. These receptors, in contrast to RAGE, are demonstrated to show both positive and negative effects on human health. They are known for their cardioprotective effects in disrupting cardiomyocyte apoptosis, whereas their high level in serum is characterized as an indicator of elevated mortality in old people [24]. The sRAGE can act as decoy receptors for binding RAGE ligands, which prevents them from attaching with membrane-bound RAGE, proving their capacity to work as protective anti-inflammatory agents. The reason for their protective capacity lies in the absence of a cytoplasmic tail in their structure. Concerning heart diseases, a study showed that sRAGE accumulation can be used as a biomarker for detecting serious coronary heart diseases such as coronary artery disease and acute coronary syndrome [25]. The minimum circulation of sRAGE during the condition of non-alcoholic fatty liver disease (NAFLD) has been established as a point of correlation between inflammation and the condition [26]. 

It has been documented that insulin resistance, AGEs, and RAGE signaling pathways (considered symptoms of diabetic complications) were all common acute symptoms of COVID-19 [27]. In a pilot study conducted to understand the effects of sRAGE and its derivatives in COVID-19 patients, it was stated that the disease can control RAGE systems, and the soluble receptors can be utilized as biomarkers and aids for treating COVID-19 [28]. 

During the treatment of AD, the RAGE molecule is targeted as it acts as a cell-surface receptor for Aβ (beta-amyloid) protein deposition in the neurons, microglia, and the epithelium lining of the brain [29]. It has been demonstrated that a high intake of dAGEs in the population is related to the prevalence of AD, and continued exposure to dAGEs can be correlated with cognitive decline and accumulation in the hippocampus zone of the brain [30]. It was confirmed through a comparative study that AGE accumulation in patients with AD can influence its development in multiple ways such as Aβ modification, mitochondrial dysfunctions, oxidative stress production, autophagy, increased permeability of the blood–brain barrier, and others [31]. An in vivo study carried out in mice to connect diabetes mellitus and AD explained that the subjects injected with AGEs demonstrated depleting memory capacity and accumulation of indicators like tau phosphorylation and APP (amyloid precursor protein) antibodies [32]. The research further suggested that diabetic patients are more susceptible to developing AD due to AGEs-related metabolism. RAGE and its ligands have also been linked to Parkinson’s disease (PD). During the condition, the cross-linking of α-synuclein proteins and their co-localization with Lewy bodies can take place agitated by the dicarbonyl precursors of AGEs [33]. Oxidative stress and inflammation resulting from mitochondrial stress have also been noted as catalysts to aggrandize PD due to AGE exposure [34]. 

Therefore, there are sufficient studies and related evidence that AGEs can be connected with various conditions and disorders as discussed earlier. So, gaining insights into the pathways and mechanisms of how AGEs affect the onset and development of the discussed conditions can prove useful in formulating preventive measures to minimize their occurrences in humans. Furthermore, there is a wide scope to conduct studies focused on studying specific pathways through which AGEs can affect human health.

## 4. Detection of AGEs

### 4.1. Sample Pre-Treatment and Extraction

#### 4.1.1. Solid-Phase Extraction (SPE)

SPE has various advantages such as lower cost, adaptability with other detection techniques, and simple procedure. It can be carried out through column construction, where the solid stationary phase is immobilized, or even commercially available cartridges are also available. SPE cartridges are more popular choices than column construction as they are available in the market pre-constructed, deployable for use on-site, and discarded easily. During a study aimed at detecting AGEs in milk powder using UHPLC-MS/MS, an MCX solid phase extraction column was used [35]. The prepared sample solution was pre-activated by 5 mL of water and 3 mL of methanol, and the column was rinsed with 3 mL of methanol and 5 mL of water, followed by elution with 6 mL of 5% ammoniated methanol. There are some drawbacks of using SPE for analyte extraction like preliminary filtration for the elimination of solid particles from the sample solution and long durations for the conditioning of the devices or sorbents.

#### 4.1.2. Reduction

It is one of the common pre-treatment and extraction techniques that is practiced for extracting AGEs from food matrices. When non-fluorescent AGEs (CML, CEL, and MG-Hs) were being extracted from fish cake samples, the samples were defatted in chloroform–acetone (1:3, *v*/*v*) mixture with centrifugation [36]. The fat-free samples were then reduced with sodium borohydride (2 mol/L) and borate buffer (0.2 mol/L, pH 9.2) for pH maintenance. The reduction process was also performed when AGEs were extracted from roasted/grilled meat products, where the sample (50 mg) was first freed from its proteinaceous constituents using hexane (5 mL), followed by centrifugation for 10 min at 4800 rpm, and the whole sequence was repeated thrice [37]. The obtained residue was dried before subjecting to reduction with sodium borohydride (1 mol/L) and sodium borate buffer (pH 9.2, 0.2 mol/L) for 8 h at 4 °C before further sample processing. As being used primarily for extracting AGEs from food samples, the reduction process can support their extraction by converting their complex chemical structures into simpler forms for easier extraction. 

#### 4.1.3. Derivatization 

Derivatization is used for altering the chemical properties of a particular analyte during a chromatographic analysis, namely, gas chromatography (GC) and high-performance liquid chromatography (HPLC). It greatly improves the detectability and performance of the chromatographic technique [38]. The derivatization process has been predominantly used for sample pretreatment before detecting AGEs and their precursors by using agents such as *O*-phenylenediamine (OPD). It was used when α-dicarbonyl precursors of AGEs, i.e., GO, MGO, and 3-DG were extracted from sausage samples for detection with UPLC-MS/MS [39]. A 5 g sample was vortexed for 10 min with 5 mL water and 5 mL acetonitrile at consecutive times followed by shaking (20 min) and membrane centrifugation to obtain a supernatant. A 500 µL aliquot of the supernatant was combined with 500 µL of 0.2% OPD for derivatization at 37 °C for 4 h and filtered through a 0.2 µm filter before analysis. OPD accomplishes its derivatizing property by dislodging the α-dicarbonyls present in the food complex by forming quinoxaline with them. 

#### 4.1.4. Dispersive Liquid–Liquid Microextraction (DLLME)

DLLE is a fast, efficient, and facile microextraction approach to the traditional LLE because of its efficient extraction and analysis sensitivity of various compounds [40]. The procedure was used for treating red ginseng products for detecting GO and MGO through a GC–MS system [41]. After derivatization with 100 µL of OPD, the sample solution was injected with a methanol/chloroform mixture (200 µL/100 µL) using a 1.5 mL syringe. The solution was hand-shaken for some time to yield a cloudy solution of water, methanol, and chloroform. The solution was centrifuged at 2500 rpm for 3 min to settle the particulate matter. The sediment phase was transferred into a glass insert vial (150 µL), and 1 µL of the surface layer was injected into the GC–MS. 

#### 4.1.5. Ultrasound-Assisted Extraction (UAE)

In UAE, ultrasound waves of frequency (>20 kHz) are used to generate mechanical energy, which is then applied to the sample. Sonication periods for treatment certainly vary depending on the sample from which AGEs are to be extracted. The treatment was utilized for the extraction of MG-H (methylglyoxal-hydroimidazolone) AGE from black tea samples, where the sonication period was 10 min [42]. The treatment was also applied when a two-step extraction process was applied to extract acrylamide, 5-HMF (hydroxymethylfurfural), AGEs, and heterocyclic aromatic amines (HAAs) from thermally processed food products [43]. The minced food samples (1 g) were placed into separate centrifuge tubes and mixed with 10 mL of hexane for defatting. The tube was shaken (1 min), ultrasonicated for 10 min, and then centrifuged for 10 min at 10,000 rpm. The hexane phase was removed and discarded, followed by repeating the process three times. The centrifuge tube was then incorporated with 10 mL of acetone and vortexed for 1 min. The tube was again subjected to ultrasonication (20 min), followed by centrifugation for 10 min at 10,000 rpm. The supernatant layer of acetone was retained for further analysis. 

### 4.2. Instrumental Techniques for the Detection of AGEs

#### 4.2.1. Liquid Chromatography (LC)

LC is a technique through which various compounds in a liquid sample are separated when brought into contact with a solid phase. However, normal LC has many drawbacks like slow elution times and complex sample preparation procedures, for which other advanced variants of it are available, like high-performance liquid chromatography (HPLC), ultra-pressure liquid chromatography (UPLC), and ultra-high-pressure liquid chromatography (UHPLC). 

HPLC is an advanced version of LC, which is modern, powerful, and popular for its routine use for the separation, identification, and quantification of components from complex matrices. The technique works on the same principle as LC, but with the advantage of the application of high pressure. Multiple detectors can be paired with HPLC for the quantification and identification of the separated analytes from the column, such as mass spectrometer (MS), UV–Visible (UV), photo-diode array (PDA), and fluorescence detector (FLD). HPLC system has been utilized for detecting AGEs like CML from samples like bread made from various gluten-free flours [44]. Various precursors of AGEs like GO and MGO have also been detected with HPLC in combination with PDA in bread samples [45]. 

Ultra-high-pressure liquid chromatography (UHPLC), a more advanced variant of HPLC, is considered an improvement over its predecessor due to higher sample throughput and less solvent consumption. It has been used for assessing AGE content in food products like roasted pork, roasted salmon patties, and plant-based burgers with detectors such as MS/MS and MS [46,47]. However, higher maintenance is required for the smaller columns used which work under high pressure in a UHPLC system. 

Ultra-performance liquid chromatography (UPLC) is a highly efficient, dynamic, and sensitive technique with lesser requirements of solvents and improved resolution, making it an environment-friendly and cost-effective alternative to HPLC. UPLC in combination with MS/MS was used for detecting AGEs in glucose-bovine serum albumin simulation and cookies to understand the inhibitory effects of rooibos (*Aspalathus linearis*) on their formation [48]. UPLC in combination with tandem Q-Exactive MS was also utilized to detect CML and CEL as a part of monitoring thermal processing parameters of bovine milk, i.e., UHT and pasteurized milk [49]. The motive of the study was to perform a quality-control-centered evaluation to determine the most optimal temperature range for the formation of CML and CEL in the mentioned milk products during their processing. It was ascertained that the temperature range of 90–100 °C was optimal for the formation of CML and CEL at the highest quantity. The work also demonstrated that AGEs can be utilized as potential biomarkers to differentiate between UHT and pasteurized milk. 

A brief comparison can be drawn between the techniques paired with various detectors to identify technique(s) that have produced low detection limits for AGEs in food samples. Through the literature surveyed for this review work, as depicted in Table 2, the lowest LODs observed were for studies that had employed UHPLC-Orbitrap-Q-Exactive-MS and UPLC-ESI-MS/MS. Even though the Orbitrap-Q-Exactive-MS detector can detect AGEs at such low levels, Orbitrap-based detectors have some drawbacks such as they are not suited for quantitative analysis and are highly costly to procure and maintain [50]. On the other hand, the minimal LOD for CML, CEL, MG-H1, and GOLD (0.15, 0.15, 1.22, and 2.44 ng/mL, respectively) were measured with the UPLC-ESI-MS/MS in comparison to the other depicted combinations (Table 2) like UHPLC-MS/MS, LC-MS/MS, and HPLC-FLD. Therefore, it can be concluded that the UPLC-ESI-MS/MS technique combination can be utilized for an effective detection and enumeration of AGEs in food matrices. 

#### 4.2.2. Gas Chromatography (GC)

GC is a chromatographical technique that is based on the characterization of volatiles and semi-volatile constituents present in samples. GC has been used for detecting volatile compounds like polycyclic aromatic hydrocarbons (PAHs) from food matrices [60]. GC has been used for detecting AGEs in food matrices and body fluid samples. However, the recent literature on the use of GC for detecting AGEs in food samples is quite limited. Employing liquid chromatographic techniques is considered more practical in contrast to GC for detecting AGEs in samples [61]. However, a headspace SPE-incorporated GC–MS-based method was developed, which claimed to achieve the lengthy derivatization process within 20 min (at 85 °C) by using triflouroethylhydrazine as the derivatizing agent for detecting GO and MGO in alcoholic beverages [62].

### 4.3. Immunological Assays for Detecting AGEs 

#### Enzyme-Linked Immunosorbent Assay (ELISA)

ELISA has been used for screening components like pathogens in food matrices like milk [63]. The initial data on food-derived AGEs were obtained through ELISA-based techniques. The immunoassay technique has been used for determining AGEs in foodstuffs like frozen chicken meatballs [64]. During the CML estimation in aged, stored, and thermally treated chicken meat, the analysis was carried out with a CML ELISA kit, and the results were expressed in mg/kg [65]. However, there are some inadequacies in the implementation of ELISA as a detection method for food samples such as the requirements of trained personnel and specialized equipment. 

## 5. Inhibition of AGEs

**Figure 5:**
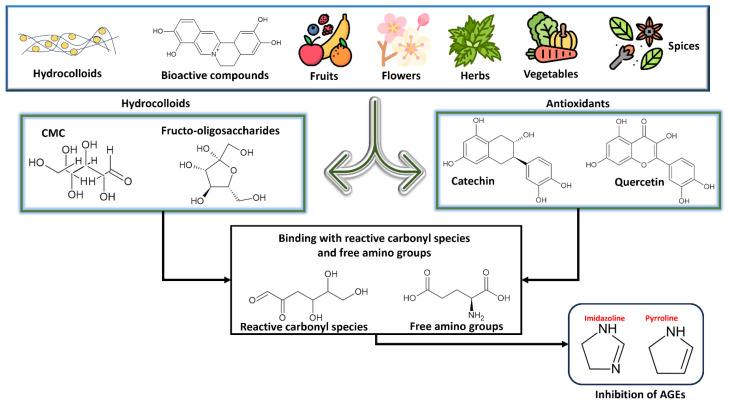
The action of various inhibitory agents in limiting the formation of AGEs.

The role of various inhibitory agents for AGE formation has been depicted in Table 3, and the mechanism of their action is in Figure 5. A thorough discussion on the use of herbs, spices, fruits, vegetables, hydrocolloids, and other advanced approaches has been discussed below. 

### 5.1. Inhibition by the Extracts of Fruits, Vegetables, and Flowers

This role of fruits in inhibiting AGEs was explored when the Chinese bayberry extract trapped α-dicarbonyl precursors of AGEs in a BSA (bovine serum albumin)-based model where 61.8% and 51.3% reductions were recorded for MGO and GO, respectively [72]. A deep eutectic solvent extract prepared from citrus peels was also studied for its suppressive activity against protein-bound and free AGEs in grilled pork meat patties [73]. The citrus peel-derived extract demonstrated a reduction of 30.5–39.8% in protein-bound AGE generation and a decrease of 19.1–68.3% in the formation of free AGEs. 

Vegetables’ inhibitory properties were employed as antiglycation agents when lotus leaves were tested for their capacity to decrease AGE formation during a study where an 80% ethanolic fraction of the leaves was taken for the experiment [74]. An ovalbumin-fructose model was constructed for conducting the AGE inhibition study, where a reduction of 55.97% was achieved through the 80% ethanolic fraction. 

*Clitoria ternatea* L. flower extract was reported to reduce the MGO-induced protein glycation and oxidative damage to DNA in a BSA system by entrapping 15–43% MGO in a concentration-dependent pattern [75]. Therefore, it is suggestible to incorporate vegetables, fruits, and edible flowers into the daily diet, especially for people consuming a meat-based diet.

### 5.2. Inhibition by Herbs and Spices

The effects of seasonings added to braised lamb during cooking were also studied on the formation of AGEs during the cooking process [76]. The powder mixture incorporated in the seasoning mixture (including light and dark soy sauce) included tangerine peel, *Fructus amomi*, *Fructus tsaoko*, myrcia, galangal, prickly ash, clove, cinnamon, and star anise. The use of spice blends significantly inhibited the formation of AGEs in the braised lamb sample by 14.13% for free AGEs and 52.10% for protein-bound AGEs upon using the spices’ 70% ethanolic extract. The study aimed at elucidating the relationship between the formation of AGEs and the four processing stages involved in producing cooked sausages and the impact of several additives on the AGE generation [77]. Various spices were signified as promoters of AGE generation, and some were found to interfere with the AGE formation. When garlic alone was added, the CEL content in the sausages increased; however, using a combination of garlic and yellow mustard slightly decreased the CML content in the sausages. Moreover, black pepper had no significant impact on CML formation while baking the sausages but significantly increased the concentration of CEL and CML when the sausages were steamed. The spices were also found to destabilize the proteins and lipids present in the cooked sausages, leading to an increased production of precursors like protein carbonyls and malondialdehyde. As the study presents contrasting inferences in comparison to the previously mentioned studies, a future thorough investigation is required to understand the effects of various spices on AGE formation in various food matrices like meat, dairy products, baked goods, etc., and how their concentration plays a role in inhibiting AGE generation. During a study, six herbal plants and their phytochemical constituents were tested for their antiglycation activity as an indication of suppressive action against AGE generation [78]. The herbs included flower buds of *Musa acuminata*, rhizomes of *Bletilla striata* and *Millettia speciosa*, dried nuts of *Amomum villosum*, roots of *Moghania philippinensis*, and stems of *Spatholobus suberectus*. *Spatholobus suberectus* depicted the highest antiglycation activity in comparison to the other used herbs, measured via bovine serum albumin–methylglyoxal and bovine serum albumin–glucose glycation models. It was unveiled through HPLC-QToF-MS/MS analysis that *Spatholobus suberectus* had 9 terpenoids and 12 polyphenols common in both water and ethanolic extracts. Compounds like saponin derivatives, apigenin, luteolin, and quercetin, identified in *Spatholobus suberectus* extracts, are known to exhibit high antiglycation and antioxidant activity. 

### 5.3. Inhibition Through Direct Addition of Bioactive Compounds

Metabolites like p-coumaric acid, an isomer of hydroxycinnamic acid, had shown a binding tendency with α-amylase, which decreased the formation of AGEs [79]. The inhibitory effects of L-ascorbic acid and quercetin against AGEs in roasted eel were also studied [80]. L-ascorbic acid was able to suppress the formation of CML and CEL by 20.36% and 27.91% and quercetin by 2.67% and 11.23%, respectively. The study credited AGE reduction in the food sample to the antioxidative effects of L-ascorbic acid and quercetin in suppressing the reactive dicarbonyl compounds, which are necessary precursors in the formation of CML and CEL. The inhibitory action of glycyrrhizic acid, liquiritin, and liquiritigenin against AGE production in thermal reaction meat flavorings was also investigated [81]. All three compounds were found to actively suppress the generation of AGEs on a dose-dependent basis. A concentration of 1.5 mL of liquiritigenin depicted the maximum inhibitory effect against CML, with a reduction of 38.69%. While for CEL, all three bioactive agents showed a good dose–effect association. At the highest dose, the reduction rates were 52.04%, 52.11%, and 61.27% for glycyrrhizic acid, liquiritin, and liquiritigenin, respectively, against CEL. 

### 5.4. Inhibition Using Hydrocolloids

The supplementation of hydrocolloids may increase the water-holding capacity of batters, which may reduce the oil absorption and formation of AGEs via the depleted rate of the Maillard reaction and reactive carbonyl compound generation [82]. This was concluded in a study conducted to examine the effects of hydrocolloids on AGE formation in deep-fried fish nuggets. Among multiple hydrocolloids tested for their AGE-reduction capacity, chitosan was identified as the AGE inhibitor [83]. When it was added to sponge cakes at a concentration of 0.5% (*w*/*w*), the documented decrease in the AGE development ranged from 30.31% to 61.22%. Carboxymethyl cellulose (CMC) also has inhibitory action against the formation of AGEs, which was determined during a study where its inhibition activity was elucidated in plant-based meat alternatives [15]. A concentration of 4% CMC addition into the plant-based meat samples inhibited the formation of CML and CEL by 75% and 83%, respectively. Furthermore, combinations of hydrocolloids and other components such as bioactive compounds might impart a complementary effect on the inhibition of AGEs. Chito-oligosaccharides (COOSs) were tested for their impact on suppressing RAGE and AGEs’ induced inflammatory responses for modulating intestinal health [84]. 

Alginic acid and pectin were studied for their inhibitory action against CML and CEL in roast fish patties, where a concentration of 0.9% of both reduced CML by 44.9% and 43.6% and CEL by 32.6% and 32.4%, respectively [85]. Polysaccharides derived from *Auricularia auricula* (wood ear fungus) (AAPs) were also tested for their potential to suppress AGE formation via a CML-induced HK-2 cellular model and an in vitro BSA-fructose model [86]. AAP was successful in preserving the proteins from oxidative damage, protecting protein sulfhydryl groups from oxidation, decreasing protein carbonylation, shielding structural changes in proteins, and mitigating the generation of β-crosslinked structures. 

### 5.5. Inhibition by Manipulation of Cooking Treatments and Storage Conditions 

The role of high-pressure processing with multiple pre-incubation temperatures was also studied for reducing the AGE content in processed milk [87]. A significant reduction was achieved in milk in comparison to the milk sample that had undergone commercial thermal processing. Additionally, gentle cooking processes as practiced in Mediterranean culinary cultures by cooking in earthenware pots take place at the temperature of 80 °C, where fluorescent AGE formation was observed to be inhibited [88]. 

Various cooking conditions and treatments were studied for their effects on AGE generation while preparing braised lamb [35]. The hot blanching treatment was found to significantly reduce the presence of free AGEs in the lamb meat samples than observed in the cold blanched group. The process of hot blanching resulted in the greater removal of precursors from the lamb samples due to a higher heating power in comparison to cold blanching. The same inferences were made when sturgeon fish patties were deep-fried and pan-fried, and the deep-fried patties demonstrated a higher accumulation of AGE content than recorded in the pan-fried patties [89]. The formation of CML during the pan-frying and deep-frying processes can be attributed to the increase in the levels of free sulfhydryl groups. 

### 5.6. Advanced Approaches to Inhibit AGE Formation

An advanced approach of plasma-activated water was employed for mitigating AGE production in roasted beef patties [90]. The plasma-activated water (PAW) used during the study was obtained using a dielectric barrier discharge plasma system. The system contained two parallel electrodes that worked under a high-voltage field to ionize air present between them. PAW treatment led to a decrease in the contents of free and bound AGEs by 23.08% and 8.42%, respectively. The presence of highly reactive species in PAW was credited with scavenging the free radicals during the roasting process, which led to the reduced lipid peroxidation and, finally, decreased occurrence of AGEs in the beef patties. 

Lactic acid bacteria (LAB) have been reported to decrease AGEs. *Lactobacillus fermentum* was recorded to inhibit the formation of CML and CEL during vinegar fermentation [91]. The inhibition rates for CML and CEL were 16.28% and 14.32% (during alcohol fermentation) and 17.07% and 14.29% (during acetic acid fermentation), respectively. In another study, *Lactobacillus fermentum* was observed to reduce the amounts of fluorescent AGEs and pentosidine by 77.22% and 51.67%, respectively, in a BSA–glucose glycation model [92].

Nanoparticles have a variety of applications such as drug delivery systems, antimicrobial agents for integration in food packaging, performance enhancers in construction materials, wastewater treatment, and many more [93]. Some recent studies have focused on utilizing nanoparticles synthesized using plant extract, i.e., green synthesized nanoparticles for alleviating the AGE presence. One study aimed at employing zinc oxide nanoparticles (ZnO-NPs) synthesized using hummingbird tree (*Sesbania grandiflora*) leaf extract [94]. The produced ZnO-NPs were able to reduce the α-amylase activity by 72% and α-glucosidase activity by 65%. Thus, they carried the capacity to reduce carbohydrate absorption from the diet, elevate glucose uptake, and prevent protein glycation. In another study, chemically synthesized ZnO-NPs and green synthesized ZnO-NPs (using *Illicium verum* extract) were compared for their pentosidine-like AGE and Vespelysine-like AGE reduction potential [95]. 

Furthermore, nanoparticles as part of nanocomposites carry a high potential for encapsulation and entrapping compounds of interest, which can enhance the reduction viability of the compounds (like antioxidants) against AGEs. Nanoparticles of lotus seedpod oligomeric procyanidins (LSOPs) were created using carboxymethyl chitosan (CMC-LSOPs-NPs) to enumerate their inhibitory effects on dAGEs liberated from the digestion of glycated casein [68]. The CMC-LSOPs-NPs were successfully able to produce a significant reduction in the release of dAGEs from glycated casein by 14.33% in the intestinal phase and by 25.76% in the gastric phase. Moreover, the antioxidant betanin was encapsulated in chitosan-sodium tripolyphosphate coated quaternary ammonium-functionalized mesoporous silica nanoparticles (betanin-CS@QAM-SNPs) to enhance and study the suppressive capacity of betanin against AGE generation in a BSA–glucose simulation and biscuit system [96]. The inhibition rate of AGEs in the BSA–glucose simulation model through betanin-CS@QAM-SNPs was 70.29%, which was contrastingly higher than of betanin alone (39.48%). The betanin-CS@QAM-SNPs were able to reduce the AGE formation by 12.5%, with the possibility of a further adsorption of AGEs formed when biscuits are digested.

## 6. Conclusions

The formation of AGEs during thermal processing and under other feasible conditions with the presence of required precursors in foods is almost unavoidable. They pose significant health risks due to contributing to conditions like neurodegenerative disorders (AD and PD), diabetes, and cardiovascular diseases. The accumulation of AGEs can take place either through dietary intake (dAGEs) or endogenously (endogenous AGEs). This highlights the need for a better understanding of their formation pathways. The formation of AGEs in food matrices is highly complex, making it a challenge to mitigate their formation, particularly in heat-processed and protein-rich food products as they undergo the Maillard reaction and chemical reactions. 

The role of detection techniques such as chromatographic techniques is important for their accurate determination in food samples. However, chromatographic techniques are highly time-consuming and require resources like solvents in large quantities. The estimation of a single analyte multiple times a day might become a laborious task to proceed with due to the high costs of operating, which limits their everyday applications. Therefore, future prospects might include the development of rapid, highly sensitive, and cost-effective NP-based kits for their on-site detection during production runs to continuously monitor AGE generation in processed food products.

The inhibition of AGEs in food products becomes a crucial task, which has been achieved through the use of natural bioactive compounds, spices, herbs, fruits, and vegetables in various studies. Even recent research works on hydrocolloids have also focused on their utilization in reducing AGE production rates in foods and simulation models. Certain advanced approaches like PAW and probiotics have also provided new avenues for inhibiting AGE production in food products. However, there is still potential in combining traditional cooking approaches with emerging inhibitory technologies/methods for minimizing the formation of AGEs in heat-processed food products like nanocomposites as carriers for thermally sensitive bioactive compounds with high antioxidant activity for reducing AGE formation during processing and, even after it, as during digestion and assimilation into the body. More research is needed in this area, which is essential to produce and establish effective regulations, formulate dietary solutions, and improve public health to mitigate the long-term risk associated with AGE exposure to humans. 

## Figures and Tables

**Table 1:** Reported diseases and disorders caused by AGEs ^1^.

Health Issue	Mechanism	Reference
Periodontitis	RAGE	[12]
Polycystic ovary syndrome	Oxidative stress and cytokines	[13]
Diabetic cardiomyopathy	Mitochondrial dysfunction in cardiomyocytes and endothelial cells	[14]
Breast cancer	RAGE induced DNA damage	[15]
Non-alcoholic fatty liver disease (NAFLD) and non-alcoholic steatohepatitis (NASH)	RAGE mediated pro-inflammatory signals produced by AGEs	[16]

^1^ AGEs, advanced glycation end products; RAGE, receptor for advanced glycation end products.

**Table 2:** Detection of AGEs in multiple food matrices ^1^.

Food Matrix	Extraction and Purification	Detection					LOD		Reference
		LPSE	Column	Mobile Phases		Detector			
				A	B				
Infant formula	Microwave-assisted acid hydrolysis	UHPLC	Syncronis HILIC (100 mm × 2.1 mm, 1.7 µm)	Water (containing 5 mM ammonium formate and 0.5% formic acid) [AGEs]	95% acetonitrile (containing 5 mM ammonium formate and 0.5% formic acid) [AGEs]	Orbitrap Q Exactive MS	**AGEs**	**ng/mL**	[35]
							Furosine CML CEL MG-H1 GOLD	4.5 19.71 12.40 4.58 12.43	
Canned meat and seafood products	Reduction, acid hydrolysis, and derivatization	UPLC	Acquity BEH amide (2.1 × 100 mm, 1.7 µm)	98% acetonitrile (containing 5 mM ammonium formate and 0.1% formic acid) [AGEs]	95% acetonitrile (containing 5 mM ammonium formate and 0.1% formic acid) [AGEs]	ESI-MS/MS	**AGEs and α-dicarbonyls**	**ng/mL**	[51]
							CML CEL MG-H1 MOLD GOLD Furosine GO MGO 3-DG	0.15 0.15 1.22 4.88 2.44 0.04 0.49 0.98 0.06	
				Water (containing 0.1% formic acid) [α-dicarbonyls]	Acetonitrile (containing 0.1% formic acid [α-dicarbonyls])				
Dairy food model	Reduction and acid hydrolysis	HPLC	-	0.1% trifluoroacetic acid in double deionized water	Acetonitrile	MS/MS	**AGEs**	-	[52]
							CML	-	
Milk powder	LLE and SPE	UHPLC	Acquity UPLC BEH C_18_ (2.1 mm × 50 mm, 1.7 µm)	Methanol with 0.1% formic acid	Water with 0.1% formic acid	MS/MS	**AGEs**	**µg/kg**	[53]
							CML CEL Pyrraline	4 4 19	
Whole-milk powder	Reduction, acid hydrolysis, and SPE	LC	Water T3 (2.1 × 150 mm, 3.5 µm)	Acetonitrile	0.1% formic acid	MS	**AGEs**	**mg/kg**	[54]
							CML CEL Pyrraline	0.044 0.039 0.070	
Roast beef patties	Reduction and acid hydrolysis	UPLC	HHS T3 (150 × 2.1 mm, 3.5 µm)	Acetonitrile	0.1% formic acid	Q-TOF-MS	**AGEs**	-	[55]
							CML CEL	- -	
Chips, crackers, and breakfast cereals	Derivatization	HPLC	Inersil ODS-3 (250 × 4.6 mm, 5 µm)	Methanol–water–acetonitrile (42:56:2, *v*/*v*/*v*)		UV-Vis	**α-dicarbonyls**	**µg/100 g**	[56]
							GO MGO	1.20 1.30	
Beer	Derivatization	UHPLC	Acquity CSH C_18_ (2.1 mm × 50 mm i.d.; 1.7 µm particle size)	0.1% formic acid in water	0.1% formic acid in acetonitrile	MS/MS	**α-dicarbonyls**	**femtomole**	[57]
							3-DG GO MGO	50 25 31	
Commercial cow, goat, and soy protein-based infant formulas	Reduction and acid hydrolysis	UPLC	BEH C_18_ column (100 × 2.1 mm, 1.7 µm)	5 mM nonafluoropentanoic acid	Acetonitrile	MS/MS	**AGEs**	-	[58]
							CML CEL MG-H1	- - -	
Beef meatballs	Reduction, acid hydrolysis, and derivatization	HPLC	ODS-80 TM (25 cm × 4.6 mm, 5 µm)	Acetate buffer/acetonitrile (90:10, *v*/*v*)	Acetonitrile	FLD	**AGEs**	**µg/mL**	[59]
							CML	0.64	

^1^ AGEs, advanced glycation end products, LPSE, Liquid Phase Separation Systems, UHPLC, ultra-high-pressure liquid chromatography; UPLC, ultra-performance liquid chromatography; HPLC, high-pressure liquid chromatography; MS, mass spectrophotometry; MS/MS, mass spectrophotometer/mass spectrophotometer; ESI, electron spray ionization; TOF, time of flight; FLD, fluorescence detector; CML, N-*ϵ*-carboxymethyl lysine; CEL, N-*ϵ*-(1-carboxyethyl) lysine; MGO, methylglyoxal; MG-H1, MGO-derived hydroimidazolone.

**Table 3:** Inhibition of AGEs by various inhibitory agents ^1^.

Inhibitory Agent	Food Matrix	Targeted AGEs	Concentration	Reduction Levels (%)	Reference
Resveratrol	Baked milk and baked yogurt	CEL and Furosine	1 and 10 µmol/L	25 and 40.97	[66]
Spinach microgreen	Bread	Fluorescent AGEs	80 g (low-level spinach or LSB) and 120 g (high-level spinach or HSB)	**Upper crust**: 29.32 (LSB) 57.18 (HSB) **Bottom crust**: 34.90 (LSB) 43.11 (HSB)	[67]
Green tea extract	Air-fried cupcakes	Fluorescent AGEs	0.058 g and 0.094 g (in terms of epigallocatechin-3-gallate (EGCG)	43.0 and 51.7	[68]
Conjugate of β-alanine-histidine nanodots and superparamagnetic NPs	Milk system	CML, CEL, and Pyrraline	-	31.0, 41.4, and 66.9	[69]
Banana blossom powder	Chocolate brownies	Fluorescent AGEs, protein-bound CML and CEL, and free CML and CEL	2.0 (*w*/*w*)	51.62, 25.92, 24.85, 33.45, and 21.01	[70]
Catechin–iron	Watermelon vinegar	CML and CEL	-	70.30 and 28.32	[71]

^1^ CEL, N-*ϵ*-(1-carboxyethyl) lysine; CML, N- *ϵ*-carboxymethyl lysine; AGEs, advanced glycation end products.

## Data Availability

Data are available within the article.
